# Adaptations of the balloon analog risk task for neuroimaging settings: a systematic review

**DOI:** 10.3389/fnins.2023.1237734

**Published:** 2023-09-18

**Authors:** Charline Compagne, Juliana Teti Mayer, Damien Gabriel, Alexandre Comte, Eloi Magnin, Djamila Bennabi, Thomas Tannou

**Affiliations:** ^1^UR LINC, Université de Franche-Comté, Besançon, France; ^2^CIC-1431 INSERM, Centre Hospitalier Universitaire, Besançon, France; ^3^Centre Département de Psychiatrie de l’Adulte, Centre Hospitalier Universitaire, Besançon, France; ^4^Plateforme de Neuroimagerie Fonctionnelle Neuraxess, Besançon, France; ^5^CHU Département de Neurologie, Centre Hospitalier Universitaire, Besançon, France; ^6^Centre Expert Dépression Résistante Fondamentale, Centre Hospitalier Universitaire, Besançon, France; ^7^CIUSS Centre-Sud de l’Ile de Montréal, Centre de Recherche de l’Institut Universitaire de Gériatrie de Montréal, Montréal, QC, Canada

**Keywords:** balloon analog risk task, functional, neuroimaging, decision making, taking, electroencephalography, functional magnetic resonance imaging

## Abstract

**Introduction:**

The Balloon Analog Risk Task (BART), a computerized behavioral paradigm, is one of the most common tools used to assess the risk-taking propensity of an individual. Since its initial behavioral version, the BART has been adapted to neuroimaging technique to explore brain networks of risk-taking behavior. However, while there are a variety of paradigms adapted to neuroimaging to date, no consensus has been reached on the best paradigm with the appropriate parameters to study the brain during risk-taking assessed by the BART. In this review of the literature, we aimed to identify the most appropriate BART parameters to adapt the initial paradigm to neuroimaging and increase the reliability of this tool.

**Methods:**

A systematic review focused on the BART versions adapted to neuroimaging was performed in accordance with PRISMA guidelines.

**Results:**

A total of 105 articles with 6,879 subjects identified from the PubMed database met the inclusion criteria. The BART was adapted in four neuroimaging techniques, mostly in functional magnetic resonance imaging or electroencephalography settings.

**Discussion:**

First, to adapt the BART to neuroimaging, a delay was included between each trial, the total number of inflations was reduced between 12 and 30 pumps, and the number of trials was increased between 80 and 100 balloons, enabling us to respect the recording constraints of neuroimaging. Second, explicit feedback about the balloon burst limited the decisions under ambiguity associated with the first trials. Third, employing an outcome index that provides more informative measures than the standard average pump score, along with a model incorporating an exponential monotonic increase in explosion probability and a maximum explosion probability between 50 and 75%, can yield a reliable estimation of risk profile. Additionally, enhancing participant motivation can be achieved by increasing the reward in line with the risk level and implementing payment based on their performance in the BART. Although there is no universal adaptation of the BART to neuroimaging, and depending on the objectives of a study, an adjustment of parameters optimizes its evaluation and clinical utility in assessing risk-taking.

## 1. Introduction

Decision making is a fundamental process in our daily lives. While some of our decisions have trivial consequences, others are associated with risks and can alter the course of our lives. Decision making requires an assessment of the positive or negative consequences resulting from the choice made, especially when the decision involves risk (Leota et al., 2021). Thus, it depends on the perception of risks and possible benefits. Indeed, prior research has demonstrated that risk assessments are needed during decision making to evaluate the risk–benefit ratio (Blais and Weber, 2006). Inadequate analysis of choices and excessively risky approaches can yield to poor decision making and have deleterious consequences for health and safety. Risk-taking behavior has been evaluated with various instruments, such as self-report measures with the Domain-Specific Risk-Taking scale (Blais and Weber, 2006), neuropsychological assessments with the evaluation of executive functions, or behavioral laboratory measures (Palmer and Harmell, 2016). The Balloon Analog Risk Task (BART), is a computerized behavioral paradigm frequently used to assess risk-taking behavior (Lejuez et al., 2002). In the BART, each pump can either inflate the balloon and increase the reward or lead to the balloon bursting and losing all rewards for that trial. The monetary reward can be collected in a permanent bank. Thus, a larger balloon is associated with a higher reward but also a higher probability of bursting. Consequently, the challenge in the BART is to balance the potential increase in reward by pumping the balloon with the risk of losing all rewards. In the initial BART, each balloon is inflated by repeatedly pressing the computer mouse or keyboard and the outcome probabilities are implicit. The objective of the BART is to obtain the highest possible reward. The average adjusted number of pumps represents risk-taking propensity and corresponds to the average number of pumps before a participant successfully banks their reward. A high adjusted score indicates high-risk behavior.

In the initial paradigm, each pump is worth $0.05 and there are a total of 90 balloons (30 for each color: orange, yellow, and blue). Each balloon color is associated with a specific explosion probability. The probability distribution ranges from 1 to 8 for orange balloons, 1 to 32 for yellow balloons, and 1 to 128 for blue balloons. Thus, the probabilities of exploding on the first pump are 1/8, 1/32, and 1/128, respectively, and increase linearly with each subsequent pump until the last pump, at which point the probability of an explosion is 1.00. In blue balloons, the average adjusted number of pumps is significantly correlated with self-report measures of personality traits, including sensation seeking and impulsivity, as well as real-world risk behaviors, including the domains of substance use (i.e., smoking), gambling, unsafe sex, and delinquency (i.e., stealing) (Lejuez et al., 2002, 2003a,b, 2004). Based on these results, the BART is often used to measure the risk-taking propensity of individuals, both in healthy and clinical samples, especially with its blue balloon characteristics. Since its initial development, the BART paradigm has varied according to the objectives of experiments and their environmental constraints, such as the number and type of trials, the number of pumps, the reward per pump, or the probability of explosion. However, the most common parameters in these various behavioral BART paradigms, as demonstrated by the blue balloons in the initial paradigm, included 30 trials, 128 possible pumps, a probability of 1/128 at the first pump, and a $0.05 reward per pump (White et al., 2008; Rose et al., 2014; MacLean et al., 2018).

Despite the BART’s popularity, its ecological validity as a behavioral measure of risk taking remains controversial. While a majority of articles found links between risk-taking behavior in the BART and real-world risk behaviors (substance abuse: alcohol, tobacco, drug) and gambling (Lejuez et al., 2002, 2003b; Aklin et al., 2005; Bornovalova et al., 2005; Hunt et al., 2005; Crowley et al., 2006; Lejuez et al., 2007; Skeel et al., 2008; Bishara et al., 2009; Bornovalova et al., 2009; Ledgerwood et al., 2009; MacPherson et al., 2010; Mishra et al., 2010; Swogger et al., 2010; Coffey et al., 2011; Krmpotich et al., 2015; Hobkirk et al., 2019), some other experiments suggested low associations. Similarly, some experiments identified positive (Reddy et al., 2014) and negative (Dominguez, 2011) associations with symptom severity, whereas no association was found in another experiment (Cheng et al., 2012). Previous research on risk-taking behavior has shown that sensation seeking and impulsivity are the main personality characteristics involved in the development of real-world risk behaviors (Reynolds et al., 2006; Zuckerman, 2007). A meta-analysis focused on the relation between personality such as impulsivity and sensation seeking, and the risk-taking in the BART (Lauriola et al., 2014). Sensation seeking is considered one of the main determinants of real-world risk behaviors during adolescence. Despite the relation between sensation seeking and real-world risk behaviors, some studies found the expected positive relationship (Lejuez et al., 2002, 2005, 2007; Pleskac, 2008; MacPherson et al., 2010) while some other studies did not find it (Lejuez et al., 2003b, 2007; Aklin et al., 2005; Benjamin and Robbins, 2007; Killgore et al., 2008). Several studies showed that the impulsivity trait is a reliable predictor of risk behaviors, such as drug abuse, risky driving, unprotected sex, and problem gambling (Chambers et al., 2003; Dahlen et al., 2005; de Wit, 2009). Similar to sensation seeking, while a relationship between impulsivity and risk taking in the BART was found in some studies (Moeller et al., 2001; Hopko et al., 2006; Holmes et al., 2009; Tull et al., 2009), other experiments did not find such an association (Hunt et al., 2005; Reynolds et al., 2006; Pleskac, 2008; Skeel et al., 2008; Bornovalova et al., 2009; Romer et al., 2009; Cyders et al., 2010; Marini and Stickle, 2010). However, three recent studies suggest an indirect relationship between impulsivity and risk-taking behavior (Hüpen et al., 2019; Teti Mayer et al., 2021; Henn et al., 2023). Thus, the literature is controversial about the link between BART and the symptom severity, impulsivity and sensation-seeking. The different results between the studies can be explained by the lack of homogeneity between the studies in terms of: (i) the population studied (healthy or clinical sample; gender; age); (ii) the conditions under which the BART was administered (stress condition, sleep deprivation); (iii) the characteristics of the BART (design, number of balloons, probability of bursting and its consequences). Previous researches demonstrated that risk-taking behavior differs according to the pathology, gender and age of the sample studied (Cauffman et al., 2010; Gowen et al., 2019; Hall et al., 2023). Similarly, the conditions under which BART was administered (depending on the aim of the study) and the parameters of the paradigm may have influenced participants’ risk-taking behavior (Gardner and Steinberg, 2005; Kessler et al., 2017; Henn et al., 2023).

Although the BART has been used in many studies in its initial version paradigm, it has limitations that may hinder the usefulness of the collected data. Indeed, previous research discussed methodological problems and identified four main shortcomings: biased scores of the average adjusted number of pumps indicating risk-taking propensity, confusion of risk with the expected value, a lack of distinction between uncertainty and risk during the early trials, and poor decomposability into adaptive and maladaptive risk behavior (Schonberg et al., 2011; De Groot and Thurik, 2018; De Groot, 2020; Canning et al., 2021). These methodological shortcomings may lead to interpretational problems, such as false-positive and false-negative results. However, these limitations in assessing risk-taking propensity can be resolved by different solutions depending on the research objectives.

In addition to behavioral studies, the BART has been used in neuroimaging to assess the brain networks underlying risk taking. The BART adapted for neuroimaging is based on the same principle, with the presentation of a balloon that must be inflated to obtain a reward. Nevertheless, these paradigms have been adapted for neuroimaging in order to obtain a sufficient number of trials, to limit motor activity that affects signal quality, and to limit the duration of the run.

Nowadays, several techniques of neuroimaging exist to study brain activity in different ways. For example, Electroencephalography (EEG), an electrophysiological technique, permits the analysis of the brain’s electrical activity with high temporal resolution (Beniczky and Schomer, 2020). Magnetoencephalography (MEG), another neuroimaging technique with strong temporal resolution, records the magnetic fields resulting from the joint activity of several thousand neurons (Schnitzler and Hirschmann, 2012). By using functional magnetic resonance imaging (fMRI) (Amaro and Barker, 2006) or near-infrared spectroscopic imaging (fNIRS) (Wilcox and Biondi, 2015), a mapping of the brain can be obtained with high spatial resolution. These two techniques record cerebral activity associated with hemodynamic phenomena by detecting local changes in blood flow. The fMRI measures the local ratio between oxyhaemoglobin and deoxyhaemoglobin, resulting in the appearance of a magnetic signal, whereas fNIRS measures changes in the absorption of infrared light between oxyhaemoglobin and deoxyhaemoglobin (Cuccia et al., 2009; Glover, 2011). As each neuroimaging technique measures brain activity differently, the use of BART during combined explorations would provide a better understanding of the neural mechanisms associated with decision-making under risk in terms of temporality and localization.

As the reliability of the BART risk-taking assessment remains controversial, a meta-analysis was conducted on the brain networks underlying risk-taking during the BART (Wang et al., 2022). The authors utilized a combination of activation likelihood estimation and connectivity analysis to identify the involvement of the reward, salience, and cognitive control networks in the BART. These findings confirm the usefulness of the BART in assessing risk-taking in neuroimaging, providing insight into the central brain networks involved.

One challenge in identifying brain networks is that the original BART paradigms used in functional neuroimaging need to be adapted due to specific recording constraints associated with each neuroimaging technique. As a result, there is a large variability of paradigms in the literature, significantly limiting the ability to explore the common brain networks involved during the BART (Rao et al., 2008; Euser et al., 2011; Mussel et al., 2015; Kiat et al., 2016).

To adapt the BART paradigm to neuroimaging techniques, the most appropriate parameters must be identified to respect environmental constraints related to neuroimaging and limit biases in the assessment of risk-taking behavior related to the research question. Considering the increasing use of neuroimaging techniques, the main goal of the present review was to describe the different BART parameters, which aimed to, first, address adaptation constraints of functional neuroimaging techniques and, second, minimize the error in approximating risk-taking profiles.

In this comprehensive literature review, the quality and utility of each BART parameter were explored to identify the most appropriate BART paradigm for neuroimaging. This study proposed several solutions for adapting BART to neuroimaging and for addressing each of BART’s shortcomings previously described. The presentation of an optimal paradigm will guide researchers in adapting and/or improving the BART to their research objectives owing to time and instrument limitations, and to obtain reliable data on risk-taking propensity.

## 2. Methods

We conducted a systematic review of the literature on BART adaptations in neuroimaging settings. Systematic reviews aim to include, according to a rigorous and reproducible methodology, all articles meeting specific inclusion and exclusion criteria in order to establish a global overview of the literature on a given subject.

### 2.1. Search strategy

This comprehensive literature search was implemented, according to PRISMA guidelines, on July 5, 2023, on the PubMed, MEDLINE and PsycInfo database (Shamseer et al., 2015). The studies were further considered according to the following inclusion criteria: (i) the study was conducted on a clinical and/or non-clinical sample, (ii) the BART was applied to a neuroimaging technique, and (iii) the study was published in a peer-reviewed journal in English. Articles were excluded on the basis of the following criteria: the study was a case report, commentary, short communication, review, meta-analysis, protocol, or abstract or did not perform the BART in a functional neuroimaging setting.

The search keywords included (“Balloon Analog Risk Task” OR “BART”) AND (“EEG” OR “fMRI” OR “fNIRS” OR “PET” OR “MEG”). The various neuroimaging techniques were identified by their classic abbreviated form, their full name, and intermediate forms. For example, the keywords used for “fMRI” included “fMRI OR “functional MRI” OR “functional Magnetic Resonance Imaging.” The search did not include restrictions concerning the date of publication. Two authors (CC and JTM) independently conducted this review and compared their samples for internal consistency. All discrepancies between reviewers were solved by a third and blind review carried out by one of the author (TT), based on the inclusion and exclusion criteria.

**Figure 1:**
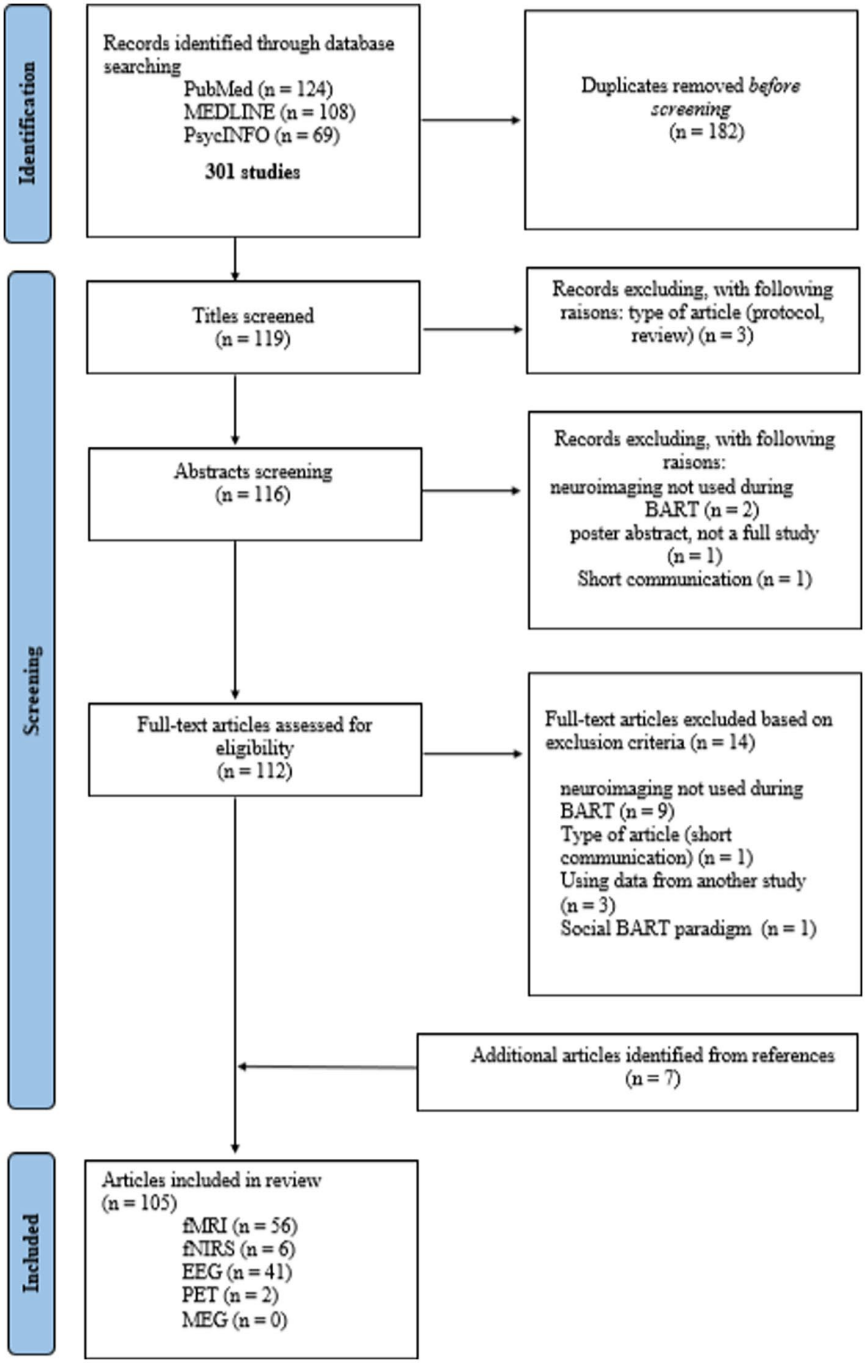
PRISMA diagram of the stages in the selection process (identification screening, inclusion) of the studies using BART during neuroimaging.

After duplicates were removed, the articles were first selected on the basis of their title, then the abstract to ensure that the studies included the BART in neuroimaging and not only in behavioral. Next, a full-text reading assessed the inclusion criteria. Finally, the references of each included article were screened following the same steps, which allowed for the identification of additional studies. A PRISMA diagram of the search process and article selection is detailed in Figure 1.

### 2.2. Extraction of information

An extraction grid was used to analyze the selected articles. This extraction grid was constructed on the basis of studies that have used BART in behavioral analysis to identify all the parameters that may impact the participant’s behaviors (Lejuez et al., 2002, 2003a,b, 2005, 2007). The data of interest were neuroimaging technique, participant data (age, gender, laterality, healthy or clinical sample), and all characteristics of the BART paradigm (design, delay between each pump and trial, number of trials, type of trial, number of possible pumps, duration of the paradigm, reward per pump, probability of explosion and its consequences, assessment of risk-taking propensity, and reward for participation). All BART parameters were studied in this review.

### 2.3. Data synthesis

To present the results in a synthetic way, the extracted data were grouped according to two modalities.

First, we provided a general and brief description of the overall results of our research strategy (number of publications, imaging technique, and subjects). Second, we presented the characteristics of the BART, which vary according to the constraints of neuroimaging techniques or the objectives of the studies.

### 2.4. Ethics statement

Our systematic review used exclusively published data and did not involve any direct interactions with human subjects. It was therefore exempt from institutional review board approval.

## 3. Results

### 3.1. Selection of publications for inclusion

The search initially yielded a total of 301 studies, from which 119 unique articles were extracted after removing duplicates. Three articles were excluded from screening based on their titles: two were protocol studies, and the third was a literature review. Four studies were also excluded from abstract screening. Two of them did not use the BART during neuroimaging recording, and one study was a short communication. The fourth article, using BART during MEG technique, was a poster abstract, the study has not been published. After screening titles and abstracts, 112 articles were selected for full-text review. Out of the 112 articles, 14 were excluded as they did not meet the eligibility criteria. Reasons for exclusion included: neuroimaging not being used during BART in nine studies, one study being a short communication, three studies using data from another study, and one study using a social BART paradigm that differed significantly from the initial BART. Based on an analysis of the list of references in the selected studies, 7 additional articles were added. Thus, this review encompassed 105 articles, involving a total of 6,879 subjects. The 105 articles referred to four distinct neuroimaging techniques: 56 fMRI, 41 EEG, 6 fNIRS, and 2 PET.

Among the fMRI articles, 32 were on healthy volunteers and 24 involved a clinical sample suffering either from Schizophrenia (Potvin et al., 2018; Tikàsz et al., 2019; Purcell et al., 2023), Parkinson’s disease (Rao et al., 2010), depressive disorders (Gao et al., 2021; Ji X. et al., 2021), bipolar disorders (Ji S. et al., 2021), traumatic brain (Chiu et al., 2012), attention-deficit/hyperactivity (Dir et al., 2019; Minchul and Jiwon, 2021), disruptive behavior disorders with histories of suicidal ideation (Dir et al., 2020), type 1 diabetes (Jorge et al., 2022), internet gaming disorder (Qi et al., 2015, 2016) or polysubstance users or dependencies such as alcohol, drug, or tobacco abuse (Bogg et al., 2012; Claus and Hutchison, 2012; Galván et al., 2013; Kohno et al., 2014; Hulvershorn et al., 2015; Forster et al., 2016, 2017; Claus et al., 2018; Burnette et al., 2020; Raymond et al., 2020).

Among the 6 articles in fNIRS recording, 5 studies were on healthy volunteers and one study on a sample suffering from opioid abuse (Huhn et al., 2019).

Concerning EEG studies, 32 were on sample healthy volunteers and 9 involved a sample suffering from alcohol abuse (Fein and Chang, 2008; Fuentemilla et al., 2013; Lannoy et al., 2017; Sehrig et al., 2019, 2020), anxiety (Nash et al., 2021), substance abuse (Euser et al., 2013; Zhong et al., 2020), and major depressive disorder (Fan et al., 2021).

Among the two PET studies, one combined fMRI and PET in healthy volunteers (Kohno et al., 2014), and one study included alcohol-dependent sample (Zorick et al., 2022).

### 3.2. Behavioral paradigms

From the initial paradigm, several parameters have been modified.

#### 3.2.1. General design

All paradigms in neuroimaging use a common base, with the presentation of a realistic image of a balloon or a circle. Among the articles, four paradigm types were identified.

Like the initial paradigm design (Lejuez et al., 2002), the most common paradigm presented an uninflated balloon, which grew in size with subsequent pumps, and accumulated earnings. Under this paradigm, participants had to press a key or their mouse to manually pump the balloon without time restrictions for decision making. This first paradigm was proposed in 93 articles (54 fMRI, 31 EEG, 6 fNIRS, and 2 PET). In this paradigm, several differences were found in the parameters used, such as the number of trials and possible pumps, type of trial, the duration of the BART, the probability of explosion, and reward conditions.

The second paradigm, used in 9 studies (7 EEG, 2 fMRI), presented an automatic response version (Euser et al., 2011, 2013; Yau et al., 2015; Hoffmann et al., 2018; Bernoster et al., 2019; Freeman et al., 2020; Morie et al., 2021; Yakobi and Danckert, 2021; Poudel et al., 2022). In this specific design, the number of pumps was determined at the beginning of each trial and then the valence of feedback was presented: either the size of the balloon was bigger and the money was earned or the balloon had popped and the money was lost.

The third paradigm proposed by one EEG study included inflation (gain) and compression (loss) condition. Here, either a little balloon had to be inflated with successive pumps or a large initial balloon could be compressed after each pump (Kiat et al., 2016). In the loss condition, the balloon started out initially large and had a maximum monetary reward. In this condition, the goal was for the participants to compress the balloon as much as possible using successive pumps to lose the reward.

In the last paradigm, found in only one EEG study, the beginning of the trial was associated with a monetary value and the participants could cash out before they had inflated the balloon (Mussel et al., 2015).

The variability of the BART parameters in neuroimaging studies is presented in Table 1. Details of each paradigm can be found in Supplementary Table S1.

**Table 1:** Variability of the BART parameters in neuroimaging studies.

BART’s parameters	Variable parameters	The initial BART (Lejuez et al., 2002)
Design	(i) Paradigm using an uninflated balloon, which grew in size with subsequent pumps, and accumulated earnings, (ii) automatic response paradigm (Euser et al., 2011 Yakobi and Danckert, 2021; Poudel et al., 2022), (iii) paradigm including inflations and compressions conditions (Kiat et al., 2016), (iv) paradigm where the beginning of the trial was associated with a monetary value (Mussel et al., 2015).	Paradigm using an uninflated balloon, which grew in size with subsequent pumps, and accumulated earnings.
Delay	A delay between (i) each pump, (ii) each trial (mostly EEG studies) or (iii) delay the beginning and end of each block of trials (Sehrig et al., 2019; Tikàsz et al., 2019; Raymond et al., 2020).	No delay
Number of trials	(i) Predetermined (Petropoulos Petalas et al., 2020), (ii) undetermined (Minchul and Jiwon, 2021).	90 (30 trials of each color)
Number of inflations	(i) Number of pumps possible was reduced (Xu et al., 2016; Li et al., 2020), (ii) large number of pumps possible (128) (automatic response paradigm) (Euser et al., 2011; Yakobi and Danckert, 2021; Poudel et al., 2022)	128 inflations
Duration of the BART	(i) Indeterminate depending on the speed of response of the participants (Lannoy et al., 2017) or (ii) determined with timed session (Kohno et al., 2017).	Indeterminate, depending on the speed of response of the participants
Type of balloons	(i) Several colors of balloons associated with specific probability (Kohno et al., 2015), (ii) control condition (fMRI and fNIRS studies) (Congdon et al., 2013), (iii) gains or losses conditions (Xu, 2021)	3 colors of balloons associated with specific explosion probability
Reward by inflation	(i) Same, (ii) double for each inflation (Weber et al., 2014), or (iii) increases according to the risk level (Rao et al., 2010), or several rewards conditions (one high and one low) (Xu et al., 2019).	Same for all balloon colors and inflations.
Duration of the training session	Variable across studies.	No training
Probability of explosion	(i) Uniform probability distribution [*p* = 1/(maximum - pumps+1)] (Rao et al., 2014, 2018), (ii) the probability of explosion was set to monotonically increase from 0 to 89.6% (Potvin et al., 2018), (3) probabilities of win and loss were equal throughout a trial (Euser et al., 2011; Gu et al., 2018), (iii) probability of explosion increased from 15 to 100% by intervals of 7 percentage points per level (Mussel et al., 2015).	Uniform probability distribution [p = 1/(maximum - pumps+1)]
Specific parameters to the probability of explosion	(i) No burst during the first or the first two inflations (Kóbor et al., 2015; Kardos et al., 2016; Sehrig et al., 2019, 2020), or (ii) a minimum number of pumps before cash out (Claus and Hutchison, 2012).	Burst during the first pump
Consequences of explosion	(i) The value is lost on that trial alone, (ii) the value is subtracted from the persistent bank (Burnette et al., 2020; Quan et al., 2022) or (iii) payment of participation (Xu et al., 2019, 2020a; Xu, 2021).	The value is lost on that trial alone
Information’s about probability of explosion	(i) Participants were not informed of explosion probabilities (Peng et al., 2020), (ii) participants knew that balloon colors might differing explosion distributions (Schonberg et al., 2012), (iii) the number of pumps where the balloon burst, was provided to the participants after a balloon popping (Yakobi and Danckert, 2021; Poudel et al., 2022), (iv) the maximum number of inflations was explicit (Poudel et al., 2022), or (v) the probability of explosion was explicit (Gu et al., 2018).	Participants were not informed of explosion probabilities
Assessment of risk behavior	(i) Average adjusted number of pumps and average adjusted number of pumps in trials following a negative and positive feedback (Ba et al., 2016), (ii) number or proportion of cash out and losses trials (Raymond et al., 2020 Gao et al., 2021), (iii) average number of pumps in loses trials (Huhn et al., 2019), (iv) average number of pumps in all trials (Euser et al., 2011), (v) minimum/maximum pump ratio (Dir et al., 2019), (vi) total earnings, earnings per trial or average total earnings before the balloon burst (vii) risky ratio (Lannoy et al., 2017), (viii) ratio of each participant’s total earnings to the average number of pumps per balloon (Poudel et al., 2022), maximum number of pumps (Euser et al., 2013), (x) average reaction time of each pump (Yu et al., 2016).	Average adjusted number of pumps
Reward for participation	(i) Standard compensation not depending to BART’s performance, and the amount earned (Takács et al., 2015), (ii) additional bonus in accordance BART’s performance (Yakobi and Danckert, 2021), (iii) Reward according BART’s performances (Gu et al., 2018), No reward (Zhong et al., 2020; Nash et al., 2021)	Reward according BART’s performances

#### 3.2.2. Training

In 50 studies (28 fMRI, 21 EEG, 1 fNIRS), participants received training before starting BART to familiarize them with the paradigm. The duration of the training session varied between studies, ranging from a few balloons (Wang et al., 2021) to a time session of 8 min (Fukunaga et al., 2012; Hoffmann et al., 2018).

#### 3.2.3. Delay

Owing to neuroimaging recording constraints, all paradigms included a variable randomized delay, either between each decision (inflation, cash out) and feedback (explosion, successful inflation) or between each trial. The delay permits to separate activation of the brain networks during decision-making and feedback processes.

In the fMRI studies, the delay between the decision and the feedback ranged from 0.2 s (Claus and Hutchison, 2012) to 6 s (Fukunaga et al., 2012), but random intervals in the range of 1–2.5 s were most frequently used. In this context, 26 studies used a small cue that changed from red to green with a jittered time interval to control the timing of the inflation period (Rao et al., 2010; Minchul and Jiwon, 2021). In 19 studies, a fixation period ranging from 10 to 60 s was also included at the beginning and end of each run to establish a baseline activity (Tikàsz et al., 2019).

Concerning the fNIRS articles, the delay of 15 s separated each trial into five studies (Cazzell et al., 2012; Lin et al., 2014; Li et al., 2017; Huhn et al., 2019; Huang et al., 2022). Only one study included a latency of 1 and 2.5 s between each pump to separate activations (Huhn et al., 2019; Huang et al., 2022; Zhao et al., 2023).

In all EEG studies, a variable delay between 0.6 and 1.5 s was added between each pump and a delay of 10 milliseconds to 3 s between each trial. In three studies, a fixation period was presented at the beginning of the balloon (Hassall et al., 2013; Chen and Wallraven, 2017; Petropoulos Petalas et al., 2020). In 4 studies, the timing of the inflation of the balloon was controlled by a cue (Hassall et al., 2013; Sehrig et al., 2019, 2020; Petropoulos Petalas et al., 2020).

In the two PET studies, a variable delay between 1 and 3 s was added between each pump and a delay of one to 14 s between each trial.

#### 3.2.4. Number of inflations and trials

The maximum number of pumps was reduced significantly in all neuroimaging studies, except in 13 articles that retained a high number of pumps (range: 60–128 pumps). The reduction of pumps per trial allowed researchers to increase the number of trials (range: 15–500 trials per study) while preventing the task from being too lengthy (Chen and Wallraven, 2017; Gu et al., 2018).

The fMRI studies significantly reduced the maximal number of pumps per trial from 8 to 30 pumps, except in five studies where it remained high (i.e., 64–128 pumps) (Chiu et al., 2012; Lighthall et al., 2012; Hoffmann et al., 2018; Poudel et al., 2022; Hu et al., 2023). In 24 studies, two or three time sessions of 6–16 min duration were offered, and so the number of trials depended on the speed of the participants’ response. The number of trials was predetermined in 13 studies (Chiu et al., 2012; Pletzer et al., 2016; McCormick and Telzer, 2017a,b; Hoffmann et al., 2018; Rao et al., 2018; Gentili et al., 2020; Ji S. et al., 2021; Jorge et al., 2022; Poudel et al., 2022; Chen et al., 2023; Hu et al., 2023; Leota et al., 2023). One study used a small predetermined time, composed of four cycles of 30 s to pump an unlimited number of balloons (Lee et al., 2009).

In all fNIRS studies, 12 pumps were possible per trial and the number of trials was fixed at 15 or 20 trials (Cazzell et al., 2012; Huang et al., 2022).

All EEG studies significantly reduced the maximal number of pumps (range: 3–50 pumps), except for 9 studies that used the automatic response paradigm with a high number of pumps (i.e., 128 pumps). Nevertheless, 12 pumps were mainly used in the BART paradigm (Zhang C. et al., 2022; Zhang K. et al., 2022). However, unlike most fMRI studies, the number of trials was predetermined at the beginning in all EEG studies and the BART’s duration varied across participants according to the speed of response.

The two PET studies proposed time sessions of 10 min.

#### 3.2.5. Duration of the BART

Thus, the duration of the BART was not limited in EEG and fNIRS, unlike the most fMRI and all PET studies that offered timed sessions. In this context, participants had a limited time to complete a maximum of trials.

#### 3.2.6. Types of balloons

##### 3.2.6.1. Control condition

A control condition was included in 30 studies (24 fMRI, 5 fNIRS, 1 EEG) studies, whereas most EEG paradigms did not include it. These studies offered an “active” and “passive” mode. Unlike the control balloon in the passive mode, colored balloons in the active mode were associated with rewards and could explode. In the active mode, participants could choose between pump or cash out as they were being forced to inflate the balloon, until the choice disappears from the screen in the passive mode. Among these studies, 8 separated control balloons and balloons with potential rewards into distinct runs (Rao et al., 2008, 2010, 2014, 2018; Lee et al., 2009; Lighthall et al., 2012; Congdon et al., 2013; Pan et al., 2019), while 14 other fMRI studies and one PET study randomized the two types of balloons throughout the paradigm (Claus and Hutchison, 2012; Schonberg et al., 2012; Galván et al., 2013; Kohno et al., 2014, 2015, 2016, 2017; Yu et al., 2016, 2017; Lei et al., 2017; Claus et al., 2018; Burnette et al., 2020; Peng et al., 2020).

##### 3.2.6.2. Balloon colors

Several balloon colors associated with various explosion probabilities in the active mode was included in 14 fMRI and 1 PET studies, as in some behavioral BART paradigms. The reward remained the same, but the maximum number of pumps varied according to the color of the balloon. In our review, we found a paradigm that included up to four different types of balloons (Schonberg et al., 2012). Only one study included two colors of balloon but they did not differ in any other attributes (i.e., reward level, size, and the distribution of explosion probability) (Hu et al., 2023).

#### 3.2.7. Reward per pump

The type and value reward for each pump were not consistent across studies.

##### 3.2.7.1. The type of reward

In 71 paradigms, the balloons were associated with a monetary reward, while in 24 paradigms, the accumulation of points constituted the reward. Moreover, 9 studies did not specify the type of reward nor the amount of reward (Lee et al., 2009; Claus and Hutchison, 2012; Fukunaga et al., 2012; Wei et al., 2016; Claus et al., 2018; Neal and Gable, 2019; Freeman et al., 2020; Korucuoglu et al., 2020a; Ji S. et al., 2021). In 2 studies, the uninflated balloon was associated with a small initial value (Kessler et al., 2017; Petropoulos Petalas et al., 2020). In one EEG study, the beginning of the trial was associated with a high monetary value (1000€), and participants determined if they wanted to inflate the balloon or cash out before they had inflated the balloon (Mussel et al., 2015).

In addition, 2 paradigms included real and hypothetical monetary rewards (Xu et al., 2016, 2018), whereas, 4 EEG studies included a gain and loss condition (Kiat et al., 2016; Xu et al., 2020b, 2021; Xu, 2021; Zhang C. et al., 2022). In the loss condition, the amount lost represented positive feedback or each successful compression increased the participant’s reward.

##### 3.2.7.2. The value of reward

In 37 studies (24 fMRI, 11 EEG, 2 PET), the same reward for each inflation was proposed across all trials, while 18 paradigms (10 fMRI, 8 EEG) increased the reward amount proportionally to the risk (i.e., the number of pumps). In 5 studies (3 EEG, 1 fMRI, 1 fNIRS), the reward amount doubled at each inflation (Weber et al., 2014; Gu et al., 2018; Wang et al., 2019; Teti Mayer et al., 2021; Zhao et al., 2023). In 6 studies, the reward increased from 0 to 5.15 dollars, from the smallest balloon to the largest balloon (Rao et al., 2008, 2010; Li et al., 2020; Korucuoglu et al., 2020b; Ji X. et al., 2021; Hu et al., 2023). In 2 studies, the difference between each pump was outcome variance, but not expected value (i.e., the sum of each possible value multiplies its probability) (Zhang and Gu, 2018; Morie et al., 2021). In addition, the reward increased 1.6 times the amount of the previous pump in 2 studies (Zhang C. et al., 2022; Zhang K. et al., 2022). However, one EEG study offered two reward conditions, a high and a low reward (Xu et al., 2019).

#### 3.2.8. Explosion probability and consequences

The probability of explosion, its consequences and information about it varied according to the study’s objectives.

##### 3.2.8.1. Information about the explosion probabilities

In accordance with the initial BART paradigm (Lejuez et al., 2002), participants were not informed of the explosion probability. As detailed above, participants were mainly aware of the number of trials, increasing amounts, and increasing probabilities, but not of the precise probabilities (Kessler et al., 2017). In all paradigms that included several colors of balloons in active mode, participants knew that balloon colors might have differing explosion distributions, but the exact risk and reward contingencies were unknown (Schonberg et al., 2012). Participants were likewise informed about the condition (low or high reward vs. low or high explosion risk condition) in one EEG study (Xu et al., 2019). Three automatic response paradigms informed the participants about the number of pumps when a balloon burst after the balloon popped (Freeman et al., 2020; Yakobi and Danckert, 2021; Poudel et al., 2022). However, in 3 studies, the maximum number of pumps was known by participants (Euser et al., 2011; Yakobi and Danckert, 2021; Poudel et al., 2022). Then, four studies informed participants of the payoff of each pump and the winning probability (Euser et al., 2013; Gu et al., 2018; Zhang and Gu, 2018; Morie et al., 2021). In one study, participants were told that the most advantageous number of pumps was 64 across all balloons, but that individual explosion points were different between the balloons (Freeman et al., 2020). In one study, the participant was allowed to gain on 10–12 consecutive trials at the outset of each block (Crowley et al., 2009).

##### 3.2.8.2. The explosion probability

In all studies but six (Euser et al., 2013; Yau et al., 2015; Gu et al., 2018; Zhang and Gu, 2018; Korucuoglu et al., 2020a; Morie et al., 2021), the probability of explosion increased with the number of pumps. In these six paradigms (5 EEG, 1 fMRI), the explosion probability remained the same for all pumps. Thus, participants could predict the risk of each pump because the probabilities of win and loss were equal throughout a trial (Euser et al., 2011; Gu et al., 2018; Zhang and Gu, 2018).

In 57 studies (30 EEG, 26 fMRI, 1 fNIRS), the explosion probability increased with the number of pumps, with a uniform probability defined as *p = 1/(max – n + 1)*, with *max* denoting the maximum number of pumps and *n* denoting the number of prior attempts. In 21 studies (17 fMRI, 4 fNIRS), the probability of explosion over successive pumps usually increased parametrically, from 0 to 89.6 at the last pump (Wei et al., 2016; Potvin et al., 2018; Raymond et al., 2020; Nash et al., 2021).

In two studies, the probability changed during the task. In the first block, balloon explosion probabilities followed a normal distribution [i.e., *p = 1/(max – n + 1)*], than in the second block, probabilities of win and loss were equal as long as the number of pumps was not extremely low (below 10) or extremely high (above 118) (Yau et al., 2015; Morie et al., 2021). In one study, the probability of an explosion increased by 5% with each pump (Kessler et al., 2017). In the last type of paradigm, which included the possibility of cashing out before inflating the balloon, the risk of bursting increased from 15 to 100% by intervals of 7 percentage points per level (15%, 22%; 29%…) (Mussel et al., 2015).

Moreover, in 10 studies (5 fMRI, 10 EEG), no burst could occur during the first pump (Dir et al., 2019, 2020; Gentili et al., 2020; Petropoulos Petalas et al., 2020) or the first two pumps (Chiu et al., 2012; Kóbor et al., 2015; Kardos et al., 2016; McCormick and Telzer, 2017b; Sehrig et al., 2019, 2020). In addition, three fMRI paradigm included a minimum number of pumps for each balloon (2–5 pumps) in the active mode before cash out to ensure that the participant tried to earn this minimum (Claus and Hutchison, 2012; McCormick and Telzer, 2017a; Claus et al., 2018).

##### 3.2.8.3. The consequence of balloon explosion

In accordance with the initial design, the consequences of balloon explosion included a loss of reward associated with exploded balloons in all studies. The same loss was also subtracted from cumulative earnings in the permanent bank in 17 studies (9 fMRI, 7 EEG, 1 fNIRS) (Rao et al., 2008, 2010; Weber et al., 2014; Wei et al., 2016; Xu et al., 2016, 2018, 2019, 2020a; Tikàsz et al., 2019; Burnette et al., 2020; Li et al., 2020; Fan et al., 2021; Xu, 2021; Quan et al., 2022; Zhang et al., 2022b; Zhao et al., 2023) and from the cumulative earnings given for research participation in 3 EEG experiments (Xu et al., 2019, 2020a; Xu, 2021) as a penalty.

#### 3.2.9. Assessment of risk-taking behavior

The assessment of risk-taking behavior was to retain the adjusted number of pumps in 77 studies (42 fMRI, 29 EEG, 4 fNIRS, 2 PET) (Lee et al., 2009; Lei et al., 2017; Gentili et al., 2020), but other scores, such as the numbers of cash out and losses trials, number of pumps in loss trials, and total earnings, were also calculated (Lannoy et al., 2017). In 20 studies (10 fMRI, 10 EEG), the average number of pumps across all trials represented the assessment of risk taking (Fukunaga et al., 2012; Takács et al., 2015; Yakobi and Danckert, 2021).

Other scores such as the number of burst balloons, the number of balloons resulting in a gain or the number of pumps on burst balloons were also indices of risk propensity (Lannoy et al., 2017). In addition, the proportion of cash out or loss trials (i.e., number of cash out or losses trials divided by the number of total trials) was also calculated in 11 studies (Qi et al., 2015, 2016; Yu et al., 2016; Claus et al., 2018, p. 20; Gu et al., 2018; Tikàsz et al., 2019; Gao et al., 2021; Ji S. et al., 2021; Yakobi and Danckert, 2021; Ren et al., 2023; Zhao et al., 2023). However, the pump ratio was used to assess the risk-taking propensity by dividing the number of gambling decisions (pump) by the total number of decisions (pump + cash out) for each participant in only one study (Zhang and Gu, 2018). The maximum number of pumps reflected the risk-taking behavior in one study (Euser et al., 2013). In only one study, the coefficient of variation (standard deviation of adjusted number of pumps divided by the mean of the adjusted number of pumps) was also calculated (Congdon et al., 2013). In 9 studies, the reaction time of each pump was used as an indicator of risk taking (Qi et al., 2015, 2016; Yau et al., 2015; Yu et al., 2016; Forster et al., 2017; Gu et al., 2018; Petropoulos Petalas et al., 2020; Gao et al., 2021; Morie et al., 2021).

In 4 EEG studies, the mean number of adjusted pumps in trials following a positive and negative feedback was likewise calculated to show the impact of the valence of the outcomes (Ba et al., 2016; Xu et al., 2020b; Fan et al., 2021; Wang et al., 2021). In one study, the percentage to which the participants decided to inflate the balloon to a certain level (1–4) after they had made the decision to inflate the balloon to that particular level during preceding trials was calculated (e.g., participant decided to inflate the balloon to level 3, and the balloon exploded. How often is it the case that the subject inflated the balloon to level 4 again?) (Kessler et al., 2017). In 3 fMRI studies, the numbers of trials between the active and passive modes were compared (Rao et al., 2008; Claus et al., 2018; Li et al., 2020). In addition, the ratio minimum/minimum number of pumps was calculated in two studies (Hulvershorn et al., 2015; Dir et al., 2019). In one study in EEG, total pumps on cash out trials on the first five trials subtracted from total pumps on cash out trials on the last five trials was calculated (Nash et al., 2021). In one study, the risk ratio, calculated by dividing the number of risky decisions (i.e., pump on balloon explosion) by the total of number of divisions indicated the risk-taking at the BART (Gu et al., 2018). In two studies, the reward collection rate (number of win trials divided by the number of total trials) was calculated (Qi et al., 2015, 2016).

Most studies also retained the total earnings. This provided information on BART’s performance. In addition, the ratio of each participant’s total earnings to the average number of pumps per balloon was calculated in only one study (Poudel et al., 2022).

#### 3.2.10. Rewards for participation

To facilitate recruitment and participation, a monetary reward was often offered to all participants at the end of the BART, except in two EEG studies that did not have financial compensation for study participation (Zhong et al., 2020; Nash et al., 2021). The reward was variable in type of money (gift card or cash) and amplitude.

A fixed amount of reward that is, not according to BART’s performance and the amount earned was offered for participation in 61 studies (29 EEG, 28 fMRI, 3fNIRS, 1 PET). Among these 61 articles, 27 (19 EEG, 8 fMRI) studies proposed additional bonus money according to the final score of the participant on top of the predefined compensation for taking part in the study (Hassall et al., 2013; Nash et al., 2021). Only 27 studies (17 fMRI, 7 EEG, 2 fNIRS) offered a proportional reward at the performance (Lin et al., 2014; Dir et al., 2019; Pei et al., 2020; Xu, 2021).

## 4. Discussion

Since its initial version, the BART has become one of the most popular tools to assess the propensity for risk-taking of individuals, and several studies, using different versions, have adapted the BART to neuroimaging. The present review synthesized the methodological parameters and main results in neuroimaging of studies that used an adapted version of the BART paradigm.

The BART was adapted in four neuroimaging techniques: EEG, fMRI, fNIRS, and PET. There was no consensus about the uniformization of research methods addressing the neural processes of risk taking. Among these adaptations, four designs of the BART paradigms, including their variable parameters, were found.

Regarding the parameters used, some were specific to the neuroimaging techniques and others to the assessment of risk-taking behavior.

### 4.1. Specific parameters to neuroimaging techniques

The main constraint of the behavioral BART is that it generates significant movements that can alter the signal in cerebral recordings, especially in EEG. To avoid this issue, the most common parameter is to include a delay between decision and feedback periods. In most EEG studies that used the first design, the delay was included between each pump, whereas in fMRI and fNIRS studies, a delay was programmed between each trial and/or each pump according to the data analysis perspectives. Although inserting a delay presents the advantage of reducing motor interference, decisions are less emotional and more cognitive, which may (Young and McCoy, 2019) or may not impact (Crosetto and Filippin, 2013) risk taking. Indeed, motor impulsivity reduces with the delay, thus permitting the study of the inhibition response, especially the overriding of a planned or already initiated action (Bari and Robbins, 2013). As stated in the introduction section, risk taking in the BART was correlated with self-reports of trait-like impulsivity, including the Barratt Impulsiveness *Scale* (Patton et al., 1995) and the Sensation-Seeking Scale (Zuckerman and Link, 1968). A solution for this issue is to include only a delay between the feedback and the next trial (Rao et al., 2008) or include only decisions that do not involve risk taking, such as the control balloon (Teti Mayer et al., 2021). Another solution is to limit the time of the paradigm, with the number of trials varied according to the response speed of participants, to maintain emotional commitment and motivation. Setting a time limit, like in most fMRI studies, has the advantage of increasing the motivation and risk-taking of participants as they try to inflate as many balloons as possible to obtain the highest score (Ji X. et al., 2021). However, to solve issues associated with delays and respect the time constraints of imaging protocols, most studies significantly reduced the inflation capacity of the balloons. Decreasing the balloons’ capacity to between 10 and 16 pumps allowed researchers to have enough trials with a short administration, although more possible inflations would allow for more accurate risk profiles (Yu et al., 2016; Kessler et al., 2017; Di Plinio et al., 2022). Additionally, a control condition was sometimes necessary, mainly for fMRI and fNIRS studies, to differentiate the brain activity of risk taking from the baseline activity (Rao et al., 2008; Schonberg et al., 2012). Such controls facilitated the analysis of contrasts and the objectification of activations only related to decision and feedback without influencing visual and motor components. To summarize, including a delay only between each trial, setting a time limit, increasing the number of trials to between 80 and 100, and reducing the number of possible inflations between 12 and 20 allowed studies to respect the recording constraints of the neuroimaging technique.

### 4.2. Specific parameters for risk-taking propensity

As we said in the introduction section, despite the popularity and quality of BART in assessing risk taking, several methodological shortcomings on the assessment of risk-taking behavior depending on the BART parameters were identified by several papers, reviews, and meta-analysis (Schonberg et al., 2011; De Groot and Thurik, 2018; De Groot, 2020; Canning et al., 2021; Coon and Lee, 2022). Several solutions have been proposed for each shortcoming to reduce interpretational problems, including false-positive and false-negative results. Depending on the objectives of a given study, these methodological problems can be limited by the choice of appropriate experimental parameters.

#### 4.2.1. Indices for assessing risk-taking propensity

Risk tendency was calculated by the average number of adjusted pumps in the majority of paradigms, except for the automatic response version. However, a systematic review focused on the relationship between risk-taking propensity in the BART and alcohol consumption (Canning et al., 2021), suggested that the adjusted mean scores of the pumps may not be specific enough to capture the amount of information required to understand its relationship with alcohol outcomes. In the initial paradigm, the adjusted score represented the risk-taking propensity, which is the mean number of pumps for banked trials (i.e., the adjusted number of pumps) to compensate for the unadjusted biased score, including trials that exploded (Lejuez et al., 2002). When trials end with the explosion of the balloon, risk propensity appears biased because the number of pumps does not reflect the risk that participants are willing to take. In this context, the unadjusted score is biased (Pleskac, 2008; Dijkstra et al., 2022) and the inter-subject variability is reduced (Lejuez et al., 2002). Similar to the unadjusted score, the adjusted score seems to be biased because the end of trials depends on the participants’ behavior, given that the risk increases with the number of pumps (Lauriola et al., 2014). using an outcome index that is more informative than the standard average pump score. One solution is to analyze the risk-taking behavior in different ways, with a model of which decompose behavior into risk taking, response consistency, and learning (Wallsten et al., 2005); Bayesian models based on a model of behavior in terms of risk propensity and behavioral consistency (Coon and Lee, 2022); or computational models (Weller et al., 2019; Young and McCoy, 2019; Dijkstra et al., 2022). All these models provide a reliable measure of risk-taking behavior. In addition, using an outcome index that is more informative than the standard average pump score like the risk score used in other risk-taking paradigms, such as the Iowa Gambling Task (IGT; Bechara et al., 1994), allows to measure the risk-taking behavior of participants in all trials. Another solution is to modify the standard design and use the automatic response version of the BART (Pleskac, 2008). As a reminder, in this design, participants indicate the number of pumps that they intend to do at the beginning of each trial. Under this paradigm, the assessment of risk propensity is not biased because it takes into account the number of pumps in all trials. A minimum number of pumps for each balloon before cash out or balloon explosion can also be included to limit errors in risk-taking assessment, as early pumps are less associated with risk-taking behavior.

#### 4.2.2. The probability of explosion

In addition, probability explosion was modulated by linear or exponential functions across successive pumps and increased from 0 to 89.6% or 100% in all studies, except in two studies that retained the same probability of explosion for all pumps. As the value of the balloon and the probability of explosion increase with successive pumps, decision making in the BART is influenced by both risk and value, complicating the measure of participants’ risk propensity (De Groot, 2020). An equal probability between win and loss allows the study to limit the impact of the value on decision making (Gu et al., 2018). In addition, using a model explosion probability with exponential monotonic increases and a lower maximum threshold of explosion probabilities between 50 and 75% reduces stochasticity related to the BART paradigm and provides a reliable estimation of risk profiles (Di Plinio et al., 2022).

#### 4.2.3. The type of decision: risky and ambiguous

One of the limitations of the BART paradigm is the lack of clarity about the type of decision, that is, whether it is made under uncertainty or risk (De Groot and Thurik, 2018; De Groot, 2020; Di Plinio et al., 2022). Communication about the probability of explosion to participants can also affect risk-taking behavior and the type of decision. Indeed, in ambiguous conditions, the probability of reward is initially unknown, whereas it is communicated to participants in risk conditions (Wilson and Vassileva, 2018). Since explosion probabilities are unknown to participants in the initial paradigm, the behavior should be more uncertainty-driven than associated with risk during the early stages of the BART until the time participants learn more about the probabilities (de Groot and Van Strien, 2019). However, it is unclear when the type of decision changes, and the timing where decisions shift from uncertainty to risk varies across individuals and depends on the characteristics of the paradigm (Brand et al., 2006, 2007). In addition, including several balloon colors associated with specific probabilities of explosion reinforces the measurement of risk behavior but increases participants’ learning, so the time transition from decision under ambiguity and risk can improve (Claus and Hutchison, 2012; Kohno et al., 2014; Peng et al., 2020). A first solution is to add a sufficiently long training time to limit uncertainty-driven behavior, so the behavior in the early stages of the BART might be directly related to risk taking. Furthermore, the control balloon included in fMRI and fNIRS studies allows participants to estimate the maximum size and probability of winning more quickly, which reduces participants’ learning. To assess only the decision making under risk, another solution is to inform participants about the range of the number of inflations possible but not the optimal strategy (Bernoster et al., 2019; de Groot and Van Strien, 2019) or to provide explicit feedback about the number of pumps when the balloon explodes, which improves the timing of participants’ learning across trials (Yakobi and Danckert, 2021).

#### 4.2.4. Motivational and attentional components

The motivational and attentional components are important elements of BART, particularly in the performance of participants to obtain the highest score. During the BART, participants may lose motivation to achieve the highest score due to a low reward per pump. Prior research has demonstrated the importance of the motivational system in risky decision-making processes (Franken and Muris, 2006; Luna et al., 2013; Urosevic et al., 2014; Kim-Spoon et al., 2016). In that respect, the nature and magnitude of BART rewards, such as compensations for participation in research, have a major influence on the participants’ BART performance (Bornovalova et al., 2005; Xu et al., 2016, 2018, 2019). Risk taking decreases more steeply in real-reward conditions after negative feedback compared to hypothetical rewards. In addition, the punitive aspect of balloon explosions, particularly when there is a direct impact on cumulative earnings (i.e., payment for research participation), could reinforce conservative behavior and the motivation of participants (Xu et al., 2020a). Thus, increasing the reward according to the level of risk, paying participants based on their BART performance, and subtracting the value of the balloon bursting from the payment for participation, increases participants’ emotional engagement and their motivation to obtain the highest score (Schonberg et al., 2011).

#### 4.2.5. Adjustment of parameters to limit bias in the assessment risk-taking propensity

The assessment of risk-taking behavior may be affected by the design of the paradigm and its characteristics. In the initial BART design, participants may tire from inflating the balloons one pump at a time and thus limit their effort out of laziness or a desire to finish sooner (Young and McCoy, 2019). In the same way, participants might need time to learn the optimal strategy. According to our results, the automatic response version (Pleskac, 2008) seems to be the most appropriate paradigm for adapting the BART to neuroimaging and addressing the identified limitations (De Groot, 2020). Indeed, this paradigm has the following advantages: (i) it includes an automatic response paradigm limiting motor activity, tiredness, and weariness; (ii) it maintains a high balloon capacity and enough trials with a short administration; (iii) it includes a delay between decision and feedback, allowing the recording of brain activity; (iv) it provides explicit feedback to participants, which increases their learning time and limits ambiguous decisions associated with the first trials; and (v) it uses an unbiased score in all trials for more accurate risk profiles. Although the automatic response version offers many advantages, the time between decision and feedback is increased, and outcome that can impact the risk-taking behavior of participants (Young and McCoy, 2019), with decisions less impulsive and more planned (Pleskac, 2008). The motor aspect, although complicating the neuroimaging recording, is an essential part of the initial BART paradigm because it allows the impulsivity trait to be maintained in the response procedure by inflating the balloon one pump at a time.

Thus, the BART might have infinite applicative variants with a large variability of parameters, and the choice of appropriate experimental parameters is not always straightforward and intuitive. In order to generate reliable data, researchers should select the most appropriate and unbiased BART experimental settings (Di Plinio et al., 2022). An adjustment of the BART parameters would optimize its assessment of risk-taking behavior, but this needs to be tested in future research.

### 4.3. Limits

Although this review presented adaptations to the BART used in neuroimaging studies, several limitations of these paradigms should be noted.

Firstly, this study provides descriptive information on various BART parameters and does not rely on statistical tests, as is typically the case in a meta-analysis. The methodological limitations and proposed solutions are the result of synthesizing articles structured on the basis of discussions from previous systematic reviews and meta-analyses. The wide variability of paradigms limited the possibilities of comparing the impact of BART parameters on risk-taking behavioral outcomes. Although some authors have proposed meta-analyses of functional neuroimaging data on BART, we find that the variability of paradigms and the contrasting analysis approaches we have highlighted make it tricky to combine the resulting data for meta-analyses.

Secondly, the scientific quality of the articles included in the review was not homogeneous. The quality of the articles in this review varied according to the homogeneity of the sample (confounding factors of sample characteristics: age, sex, size, healthy or pathological), and the protocols used (double-blind, randomized). However, since our focus is not on studying the results of the primary studies but rather the procedures for performing the behavioral task, we considered that the quality of the articles did not significantly interfere with the characteristics of the paradigms described in the methodological section of the included studies. In fact, we examined all BART parameters to analyze the various modifications linked to adaptations for neuroimaging techniques. We have simply noted the studies in which details were not provided on certain points of analysis.

Third, this review is limited to paradigms that were published, which restricts the scope of comparisons especially for the study on the impact of various parameters on risk behavior.

## 5. Conclusion

The BART is a widely used paradigm to measure people’s risk propensity, both in behavioral and neuroimaging studies. Several versions of the BART adapted in four neuroimaging techniques were compared to determine which parameters perform best in terms of reliability and validity for measuring risk propensity. The most appropriate paradigm for adapting the BART to neuroimaging should have the following characteristics: (i) decreases the number of possible pumps (limits motor aspect and fatigue) while including enough trials for a short administration, (ii) includes a delay between the decision and outcomes for the recording of brain activity, (iii) defines a monotonic exponential probability that increases to a maximum burst probability between 50 and 75%, (iv) informs participants about the number of pumps when the balloon bursts to limit decisions under uncertainty associated with the first few trials, (v) increases the reward according to risk level, and pay participants based on their BART performance (keep participants motivated), (vi) uses an unbiased score across trials for more accurate risk profiles. Although there is no universal adaptation of the BART to neuroimaging, the adjustment of parameters optimizes its evaluation and clinical utility in assessing risk taking.

## Data availability statement

The original contributions presented in the study are included in the article/Supplementary material, further inquiries can be directed to the corresponding authors.

## Author contributions

CC, JT, DG, DB, and TT: conception or design of the work. CC and JT: selection of articles for the systematic review. CC, JT, DG, AC, EM, DB, and TT: drafting the work or revising it critically for important intellectual content and provide approval for publication of the content. All authors contributed to the article and approved the submitted version.
